# Alternative bipartite arrangements of VP1 BR1-3 drive efficient nuclear import of AAV2 capsids

**DOI:** 10.1016/j.jbc.2026.113562

**Published:** 2026-09-16

**Authors:** Mikayla Hoad, Sepehr Nematollahzadeh, Davide Cimichella, Silvia Pavan, Ciro Leonardo Pierri, Justin A. Roby, Gualtiero Alvisi, Jade K. Forwood

**Affiliations:** 1 Gulbali Institute, Charles Sturt University, Wagga Wagga, New South Wales, Australia; 2 Training Hub Promoting Regional Industry and Innovation in Virology and Epidemiology, Gulbali Institute, Charles Sturt University, Wagga Wagga, New South Wales, Australia; 3 Department of Molecular Medicine, University of Padova, Padova, Italy; 4 Department of Pharmacy-Pharmaceutical Sciences, University of Bari, Bari, Italy; 5 School of Animal and Veterinary Sciences, Charles Sturt University, Wagga Wagga, New South Wales, Australia

**Keywords:** adeno-associated virus, binding interface, importin alpha, nuclear localization, structure

## Abstract

Adeno-associated viruses (AAVs) are among the most extensively studied viral gene-therapy vectors, yet the mechanisms governing their nuclear entry remain incompletely understood. Efficient transduction requires that the AAV capsid, or its structural subunit VP1, traverse the nuclear envelope to deliver the therapeutic genome. The N-terminal region of VP1 contains three clustered basic regions (BR1-3) proposed to function as nuclear localization signals. Here, we combine cellular, biophysical, structural, and computational modelling approaches to define the nuclear import mechanism of AAV2 VP1 at molecular resolution. We show that VP1 engages the classical importin-α/β1 (IMPα/β1) pathway and binds multiple IMPα paralogs with distinct affinities. Crystallographic and mutational analyses reveal that two intact BRs are required to simultaneously occupy the major and minor binding pockets of IMPα in a bipartite configuration. Structural data indicate that mouse IMPα2 preferentially accommodates BR1 and BR3 at these sites, however, functional studies demonstrate that mutation of individual BRs does not abolish IMP binding or nuclear accumulation. This robustness arises from the ability of BR2 to flexibly engage both binding pockets, enabling the formation of alternative bipartite arrangements (BR1-BR2, BR2-BR3, or BR1-BR3). Together, these findings reveal an unexpected versatility in how AAV2 VP1 exploits the IMPα binding sites, providing a structural basis for efficient capsid nuclear import. The flexibility of BR1-3 expands the current paradigm of viral nuclear localization signal organization and suggests new strategies to fine-tune nuclear targeting AAV-based gene-therapy vectors.

Adeno-associated viruses (AAVs) were first identified as contaminants of adenovirus preparations in 1965 (1) and over the past 6 decades, have become some of the most extensively studied viral vectors for gene therapy. Several AAV-based therapeutics are now approved for clinical use, including Glybera, Luxturna, Zolgensma (2). AAVs belong to the family Parvoviridae and the genus *Dependoparvovirus* (3, 4). They are small (∼4.7 kb) single-stranded DNA viruses with a genome comprising two primary open reading frames, *rep* and *cap*, flanked by inverted terminal repeats (5). Two additional genes, *aap* and *maap*, are encoded within the *cap* region in alternative reading frames and give rise to accessory proteins (6). At least 13 serotypes have been identified from human and nonhuman primate tissues (7, 8), each exhibiting distinct biological properties that underpin their versatility as gene delivery vectors (9). Seroprevalence studies indicate that more than 80% of the human population has been exposed to at least one AAV serotype (10, 11, 12) with infections typically remaining asymptomatic (13, 14). AAV replication is dependent on co-infection with helper viruses, such as adenoviruses, herpesviruses, and papillomaviruses (15, 16, 17). AAVs are widely regarded as powerful tools for gene therapy, with the potential to treat a broad range of genetic disorders. Their ability to transduce both dividing and non-dividing cells (18, 19, 20, 21, 22), combined with relatively low immunogenicity (14) and ease of genetic manipulation (23, 24) has contributed to their success as leading gene-therapy vectors.

Over the years, extensive research has examined the mechanisms by which AAVs enter cells and traffic through the cytoplasm to the nucleus (25). However, the precise host-virus interactions that govern nuclear trafficking remain incompletely defined. AAVs are thought to traverse the nuclear pore complex (NPC) (26, 27, 28, 29) *via* specific regions of the capsid proteins (30) that interact, directly or indirectly, with nuclear import receptors (31). Despite this, neither the specific host importins (IMPs) involved nor the viral signals within that drive this process have been unequivocally identified. Given the central importance of nuclear entry for successful gene delivery, a detailed understanding of this process is critical for optimizing AAV-based therapies. We have recently characterized direct binding interactions between multiple AAV serotypes and members of the karyopherin importin-α (IMPα) family, which associate with importin-β 1 (IMPβ1), to form a trimeric complex that translocates through the NPC *via* the classical nuclear import pathway (32). Typically, IMPα recognizes short sequences enriched in positively charged residues on cargo proteins (33), known as nuclear localization signals (NLSs). These signals can be monopartite, engaging a single binding site on IMPα (34), or bipartite, comprising two basic regions (BRs) separated by a short linker that simultaneously occupy the major and minor binding pockets of IMPα (35). The AAV *cap* gene encodes three capsid proteins, VP1, VP2, and VP3, which share a common C-terminal domain but differ in their N-terminal extensions due to alternative splicing and leaky scanning during translation (36, 37, 38). VP1 contains the full N-terminal region (residues ∼1–735) (36, 37), VP2 lacks approximately the first 140 residues, and VP3 lacks the N-terminal domain entirely (36, 37). Together, these proteins assemble to form the AAV capsid. The N-terminal regions of VP1 and VP2 are thought to be sequestered within the capsid interior (39, 40) and become externalized prior to nuclear entry through pores in the virion (41, 42). Previous studies have identified BRs within the VP1/2 N-terminus as candidate NLSs (30). Consistent with this. our recent work demonstrates that three such regions, BR1, BR2, and BR3, function as NLSs and directly interact with multiple IMPα paralogs (43, 44, 45, 46, 47).

In this study, we dissect the nuclear import mechanism of VP1 from AAV2, the first identified serotype and one widely used in gene therapy owing to its established clinical efficacy (48). We show that the N-terminal BRs BR1-3 (residues 120–172) are sufficient to redirect heterologous proteins to the nucleus and to mediate high-affinity binding to specific IMPα paralogs, but not to IMPβ1 directly. Consistent with this, VP1 accumulates in the nucleus when expressed in mammalian cells but is retained in the cytoplasm in the presence of the IMPα competitive inhibitor Bimax2, confirming reliance on the classical IMPα/IMPβ1 pathway. The crystal structure of the AAV2 VP1 BR segment bound to mouse IMPα2 reveals a bipartite arrangement, with BR1 occupying the minor site and BR3 the major binding site. Unexpectedly, however, mutation of basic residues in either BR1 or BR3 reduces, but does not abolish IMPα binding or nuclear accumulation, contrary to what is typically observed for canonical bipartite NLSs. Furthermore, mutation of BR2 diminishes both IMPα binding and nuclear import, despite this region not being resolved in the VP1 BR:IMPα crystal structure. Structural and computational modelling analyses support flexible binding mode in which, upon disruption of BR1, VP1 can still engage the minor and major sites *via* BR2 and BR3, respectively, and conversely, upon disruption of BR3, *via* BR1 and BR2. Only the simultaneous disruption of two BRs fully abrogates IMPα binding and nuclear import. Collectively, these findings support a model in which AAV2 VP1 exploits an unusually plastic NLS architecture, where BR1, BR2, and BR3 can be combined in multiple bipartite configurations to ensure robust nuclear targeting.

## Results

### AAV2 VP1 BR1-3 confers nuclear targeting and binds IMPα paralogs with high affinity

We have previously shown that VP1 from AAV6 and AAV11 interacts with IMPα paralogs *via* its N-terminal BR1-3 (44, 45). We therefore hypothesized that the corresponding region in AAV2 VP1 similarly mediates nuclear targeting and IMPα binding. To test, we analyzed the subcellular localization of a green fluorescent protein (GFP) fusion containing AAV2 VP1-BR (residues 120–172) using quantitative confocal laser scanning microscopy (CLSM upon following transient expression in HEK293A cells (Fig. 1*A*). GFP alone and GFP fused to the simian vacuolating virus 40 (SV40) large tumor antigen (LTA) NLS were used as negative and positive controls, respectively. As expected, GFP alone was distributed between the nucleus and the cytoplasm (Fig. 1*B*), with a mean Fn/c of ∼1.3. In contrast, GFP-SV40 LTA NLS showed strong nuclear accumulation (Fig. 1*B*) with a Fn/c of ∼10 (Fig. 1*C*). Notably, GFP-AAV2 VP1-BR exhibited even greater nuclear accumulation than the SV40 NLS control (Fig. 1*B*), with an average Fn/c of ∼15, indicating a strong capacity to mediate nuclear targeting.

**Figure 1:**
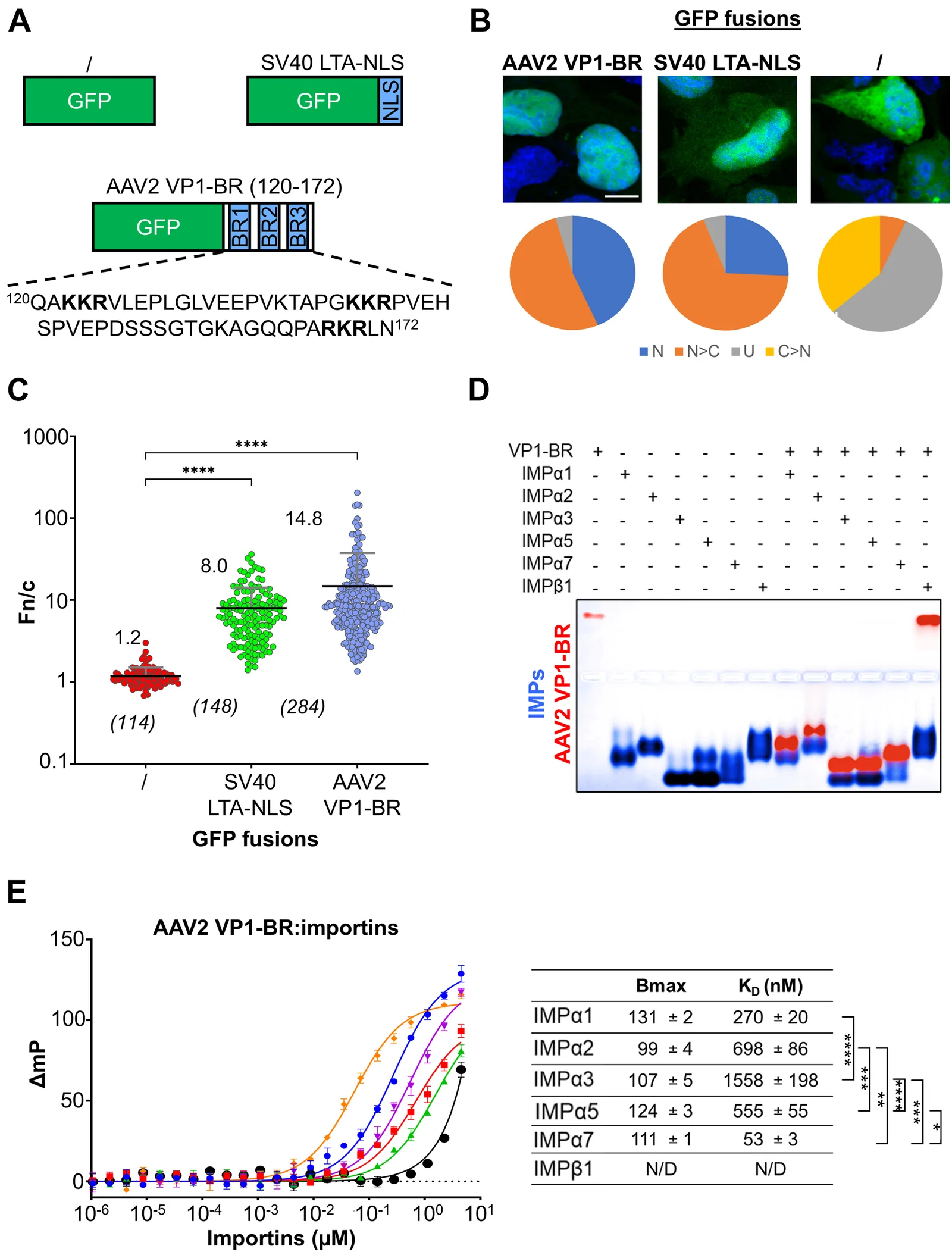
**AAV2 VP1 BR region (residues 120–172) confers nuclear targeting and binds IMPα paralogs.***A*, HEK293A were transfected with the indicated GFP-fusion proteins and incubated for 24 h prior to staining with DRAQ5 to label nuclei. Cells were fixed, mounted and analyzed by CLSM. *B*, representative merged images of cells expressing the indicated GFP-fusion proteins. Single-cell measurements were used to calculate the Fn/c ratio. Pie charts indicate the proportion of cells displaying the following phenotypes: N (Fn/c ≥ 5), N > C (2≤ Fn/c < 5), U (1< Fn/c < 2), C > N (Fn/c < 1). Scale bar = 10 μm. A total of n = 284 cells were analyzed for AAV2 VP1-BR condition, a total of n = 148 cells were analyzed for SV40 LTA-NLS condition, and a total of n = 114 cells were analyzed for GFP-only condition. *C*, quantification of nuclear accumulation. Each circle represents an individual cell measurement, *black horizontal bars* indicate the mean, and *great vertical bars* indicate standard deviation. Data are derived from three independent experiments. Statistical significance was assessed using Welch’s and Brown-Forsythe ANOVA tests (∗∗∗∗: *p* < 0.0001). *D*, electrophoretic mobility shift assay showing interaction between AAV2 VP1-BR and indicated IMPs. Proteins were visualized by Coomassie *blue* staining (*blue*), and the FITC-labelled AAV2 VP1-BR peptide was detected by fluorescence (*red*). *E*, FP analysis of binding between FITC-labelled AAV2 VP1-BR and the indicated IMPs. Data represent the mean ± SEM from three independent experiments. Statistical significance of K_D_ was assessed using Welch’s and Brown-Forsythe ANOVA tests (∗: *p* ≤ 0.05). No statistical significance indicated represents a lack of statistical significance. CLSM, confocal laser scanning microscopy; FITC, fluorescein isothiocyanate; FP, fluorescence polarization; IMP, importin.

We next assessed whether the AAV2 VP1 BR region (residues 120–172) mediates binding to IMPα paralogs (IMPα1, mIMPα2, IMPα3, IMPα5, IMPα7) and IMPβ1 using electrophoretic mobility shift assay (EMSA) (Fig. 1*D*) and fluorescence polarization (FP) (Fig. 1*E*). EMSA analysis performed using fluorescein isothiocyanate (FITC) labelled peptides in the presence or absence of recombinant IMPs, demonstrated that AAV2 VP1-BR interacts with all IMPα paralogs, but not with IMPβ1 (Fig. 1*D*). Quantitative FP measurements confirmed binding to IMPα proteins (Fig. 1*E*) and revealed distinct affinities among paralogs (Fig. 1*E*). The highest affinity was observed for IMPα7 (K_D_ ∼54 nM), followed by IMPα1 (K_D_ ∼270 nM), IMPα5 (K_D_ ∼555 nM) and mIMPα2 (K_D_ ∼698 nM), whereas binding to IMPα3 was substantially weaker (K_D_ ∼1558 nM).

### AAV2 VP1 is imported into the nucleus by the IMPα/β1 heterodimer

AAV2 VP1-BR region confers nuclear localization to GFP and mediates high affinity binding to specific IMPα paralogs but not to IMPβ1. We therefore hypothesized that nuclear import of AAV2 VP1 is mediated by the classical IMPα/β1 heterodimer. To test this, we quantitated the subcellular localization of full length AAV2 VP1 in the presence or absence of mCherry-Bimax2, a well characterized competitive inhibitor of the IMPα/IMPβ1 nuclear import pathway (49). Full-length AAV2 VP1-GFP was expressed in HEK293A cells, and its localization was analyzed by CLSM. GFP-H1E, which localizes to the nucleus independently of the IMPα/IMPβ1 pathway (50), and GFP-human cytomegalovirus (HCMV)-UL44 which undergoes IMPα/IMPβ1 dependent nuclear import (51), were used as negative and positive controls for mCherry-Bimax2 mediated inhibition respectively (Fig. 2*A*). In the absence of mCherry-Bimax2, all GFP fusion proteins exhibited predominant nuclear localization, with a Fn/c >2 in ∼100% of cells (Fig. 2*B*). Notably, AAV2 VP1-GFP accumulated in the nucleus to levels comparable to GFP-H1E and GFP-HCMV-UL44, with an average Fn/c of ∼21 (Fig. 2*C*), although slightly lower than the control proteins (Fn/c of ∼50). As expected, expression of mCherry-Bimax2 markedly reduced nuclear accumulation of GFP-UL44 (Fn/c of ∼0.3) but had no effect on GFP-H1E localization. Importantly, nuclear accumulation of AAV2 VP1-GFP was also strongly reduced in the presence of Bimax2 (Fn/c of ∼ 1.5), indicating dependence on the IMPα/β1 pathway. Collectively, these results demonstrate that AAV2 VP1 is imported into the nucleus *via* the classical IMPα/β1 heterodimer.

**Figure 2:**
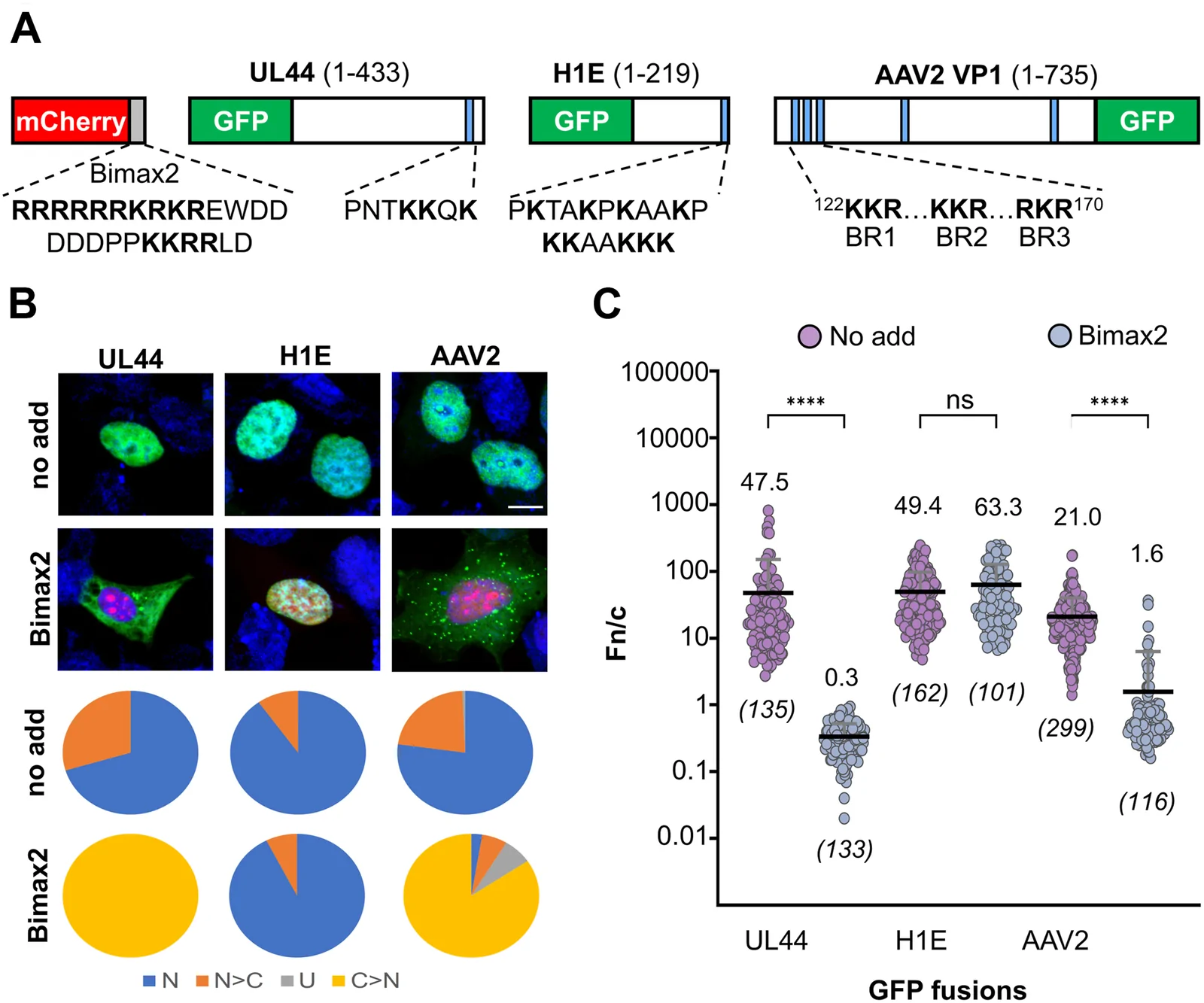
**AAV2 VP1 nuclear localization is mediated by the IMPα/β1 heterodimer.***A*, HEK293A were transfected with the indicated GFP-fusion constructs in the absence or presence of mCherry-Bimax2 for 24 h. Cells were incubated with DRAQ5 to label nuclei, fixed and mounted for quantitative CLSM analysis. *B*, representative merged images of cells expressing the indicated GFP-fusion proteins in the absence (no add) or presence of mCherry-Bimax2 (Bimax2). Single cell measurements were used to calculate the nuclear to cytoplasmic fluorescence ratio (Fn/c) for each construct. Pie charts indicate the proportion of cells displaying the following phenotypes. N (Fn/c ≥ 5), N > C (2≤ Fn/c < 5), U (1< Fn/c < 2), C > N (Fn/c < 1). A total of n = 135 cells were analyzed for UL44 no add condition, n = 133 cells were analyzed for UL44 + Bimax2 condition, n = 162 cells were analyzed for H1E no add condition, n = 101 cells were analyzed for H1E + Bimax2 condition, n = 299 cells were analyzed for AAV2 no add condition, and n = 116 cells were analyzed for AAV2 + Bimax2 condition. The scale bar = 10 μm. *C*, quantification of nuclear accumulation. Each *circle* represents an individual cell measurement, *black horizontal bars* indicate mean, and *gray vertical bars* indicate standard deviation. Data are derived from three independent experiments. Statistical significance was assessed using Welch and Brown-Forsythe ANOVA tests (∗∗∗∗: *p* < 0.0001). CLSM, confocal laser scanning microscopy.

### Crystal structure of AAV2 VP1-BR in complex with mIMPα2 reveals bipartite engagement of BR1 and BR3 at the minor and major binding sites

Our earlier structural studies provided the first insight into interactions between AAV capsid proteins and nuclear import receptors, demonstrating how different AAV serotypes engage with mIMPα2 (43, 44, 46, 47). Building on this, we determined the crystal structure of the AAV2 VP1 BR (BR1-3) in complex with mouse mIMPα2 lacking the IMPβ binding (IBB) domain (mIMPα2, mIMPα2ΔIBB) to define the molecular basis of this interaction. The mIMPα2 structure adopts the canonical α-helical architecture comprising 10 armadillo (ARM) repeats, consistent with previous reports (52) (Fig. 3*A*). The AAV2 VP1-BR:mIMPα2 complex was solved at 2.3 Å resolution and refined to R_work_/R_free_ values of 0.17/0.20. The final model comprises 426 residues of mIMPα2, 16 residues of AAV2 VP1-BR, 96 water molecules, and two glycerol molecules (Table S3). The VP1-BR peptide engages both the major and minor cargo binding sites of mIMPα2, consistent with a bipartite NLS. Interactions occur at the major site (ARM repeats 2–4, P1-P5 sites) and the minor site (ARM repeats 6–8, P1′-P3′ sites), and are mediated by 24 hydrogen bonds (17 in the major site and seven in the minor), and two salt bridges (one at each site) (Table S4). Residues within BR3 occupy the major binding site, spanning the P1-P5 positions (Fig. 3*A*), and form interactions characteristic of canonical NLS recognition. In particular, Lys169 at the P2 position, a key determinant of major site binding, forms hydrogen bonds with mIMPα2 residues Asp192, Thr155, and Gly150, and establishes a salt bridge with Asp192 (Table S4). At the minor binding site, residues within BR1 engage the P1′-P3′ positions, consistent with the classical ‘KR’ motif observed in bipartite NLSs (53, 54). Specifically, Lys123 (P1′) forms hydrogen bonds with mIMPα2, while Arg124 (P2′) participates in both hydrogen bonding and a salt bridge interaction with Glu396.

**Figure 3:**
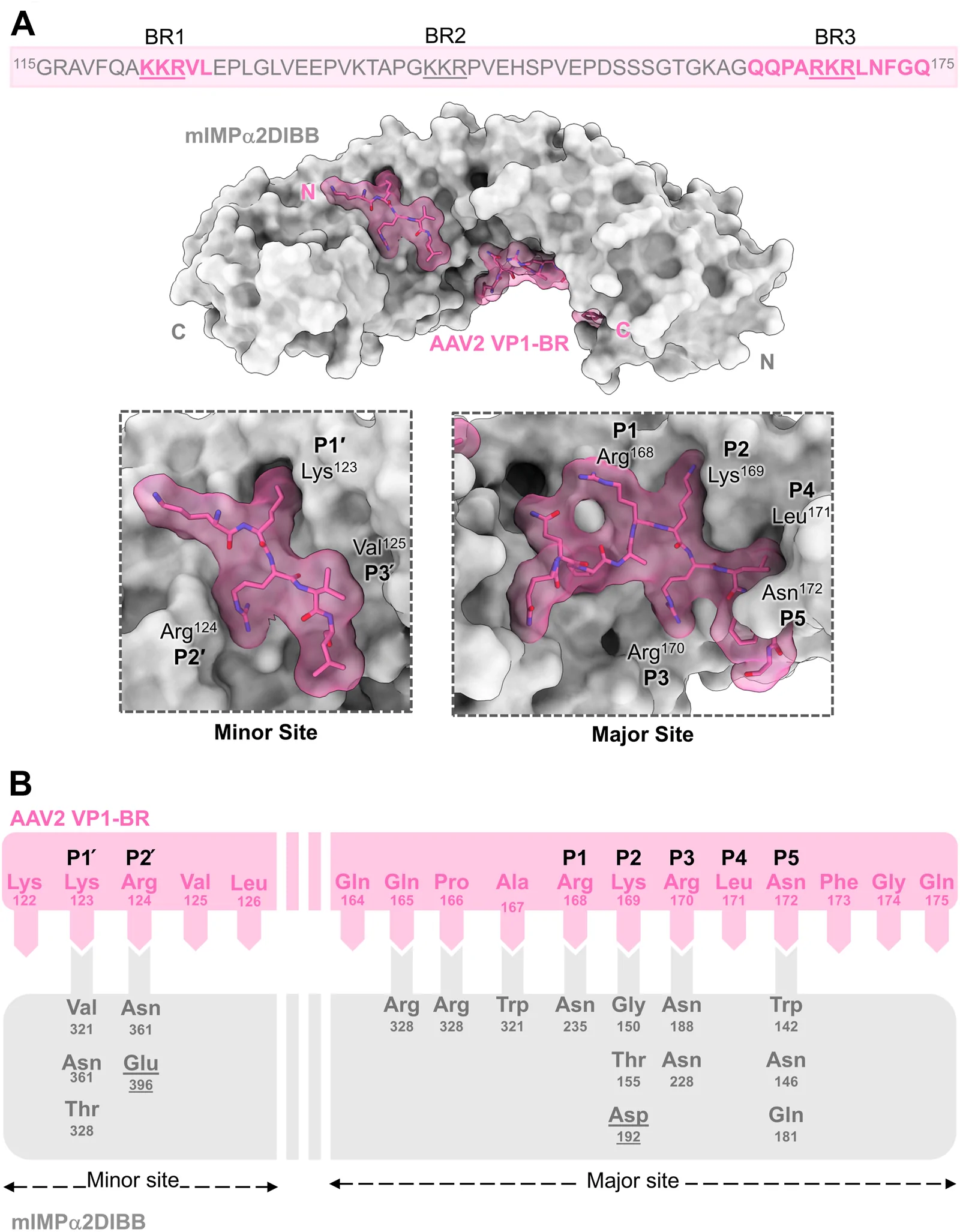
**AAV2 VP1-BR binds mIMPα2 as a bipartite NLS *via* BR1 and BR3.***A*, crystal structure of AAV2 VP1-BR (*pink sticks* and semi-transparent surface) in complex with mIMPα2 (*gray surface*) resolved at 2.3 Å. The VP1-BR sequence is shown, with residues resolved in the structure *highlighted pink* and unresolved residues in *gray*. BRs (BR1-3), defined by sequence analysis are *underlined* and annotated. *B*, schematic representation of the interactions between AAV2 VP1-BR and mIMPα2, illustrating hydrogen bonds and salt bridges (underlined). Interactions were calculated using PDBsum. The structure has been deposited in the PDB under accession code: 23QB. IMPα, importin-α; PDBsum, Protein Data Bank sum.

### Each BR within the AAV2 VP1 N-terminus contributes to nuclear targeting and IMPα interaction

The crystal structure of AAV2 VP1-BR in complex with mIMPα2 suggests that nuclear import of AAV2 VP1 is mediated by the IMPα/β1 heterodimer through recognition of a bipartite NLS formed by BR1 and BR3, which engage the minor and major binding sites of IMPα, respectively. To test this model, we assessed the effect of alanine substitution of basic residues within each BR on nuclear import of AAV2 VP1-GFP (Fig. 4, *A*–*C*) and on binding of AAV2 VP1-BR to IMPα7 (Fig. 4, *D* and *E*). Mutations targeting individual regions were generated as follows: mBR1 (K123A/R124A), mBR2 (K142A/K143A/R144A), and mBR3 (R168A/K169A/R170A). Surprisingly, substitution of either BR1 or BR3 reduced, but did not abolish, nuclear accumulation of AAV2 VP1-GFP (Fig. 4, *B* and *C*). This is contrary to the canonical requirement that both basic clusters of a bipartite NLS are essential for nuclear import activity. Notably, mutation of BR2, which is not resolved in the AAV2 VP1-BR:mIMPα2 crystal structure, also significantly reduced nuclear import (Fig. 4, *B* and *C*). In contrast, simultaneous substitutions of two BRs (mBR1+2, mBR1+3, or mBR2+3) completely abolished nuclear import, resulting in cytoplasmic localization in 100% of transfected cells (Fn/c < 1), with a mean Fn/c of ∼0.2 (Fig. 4*C*). Consistent with the cellular data, FP assays showed that individual substitutions in BR1 (K_D_ ∼518 nM, ∼10 fold reduction), BR2 (K_D_ ∼740 nM, ∼14 fold reduction), or BR3 (K_D_ ∼207 nM, ∼4 fold reduction) reduced, but did not eliminate binding to IMPα7 (Fig. 4, *D* and *E*). In contrast, double mutants involving BR1+2 resulted in binding too weak to determine a reliable K_D_, while BR1+3 and BR2+3 mutants completely abolished detectable interaction (Fig. 4, *D* and *E*). Notably, the mBR1+3 peptide, in which BR2 represents the only intact BR, failed to produce measurable binding to IMPα7 in FP assays and showed no detectable interaction in EMSA (Figs. 4, *D* and *E* and S5*E*). Thus, BR2 alone is insufficient to support high-affinity IMPα binding under these assay conditions. EMSAs using mutant peptides against multiple IMPα isoforms (IMPα1, mIMPα2, IMPα3, IMPα5, IMPα7) and IMPβ1 yielded consistent results, showing partial reduction in binding for single R mutants and complete loss of binding for double mutants (Fig. S4). These findings indicate that BR2 does not function as an independent monopartite NLS but instead contributes to IMPα recognition when paired with another functional BR.

**Figure 4:**
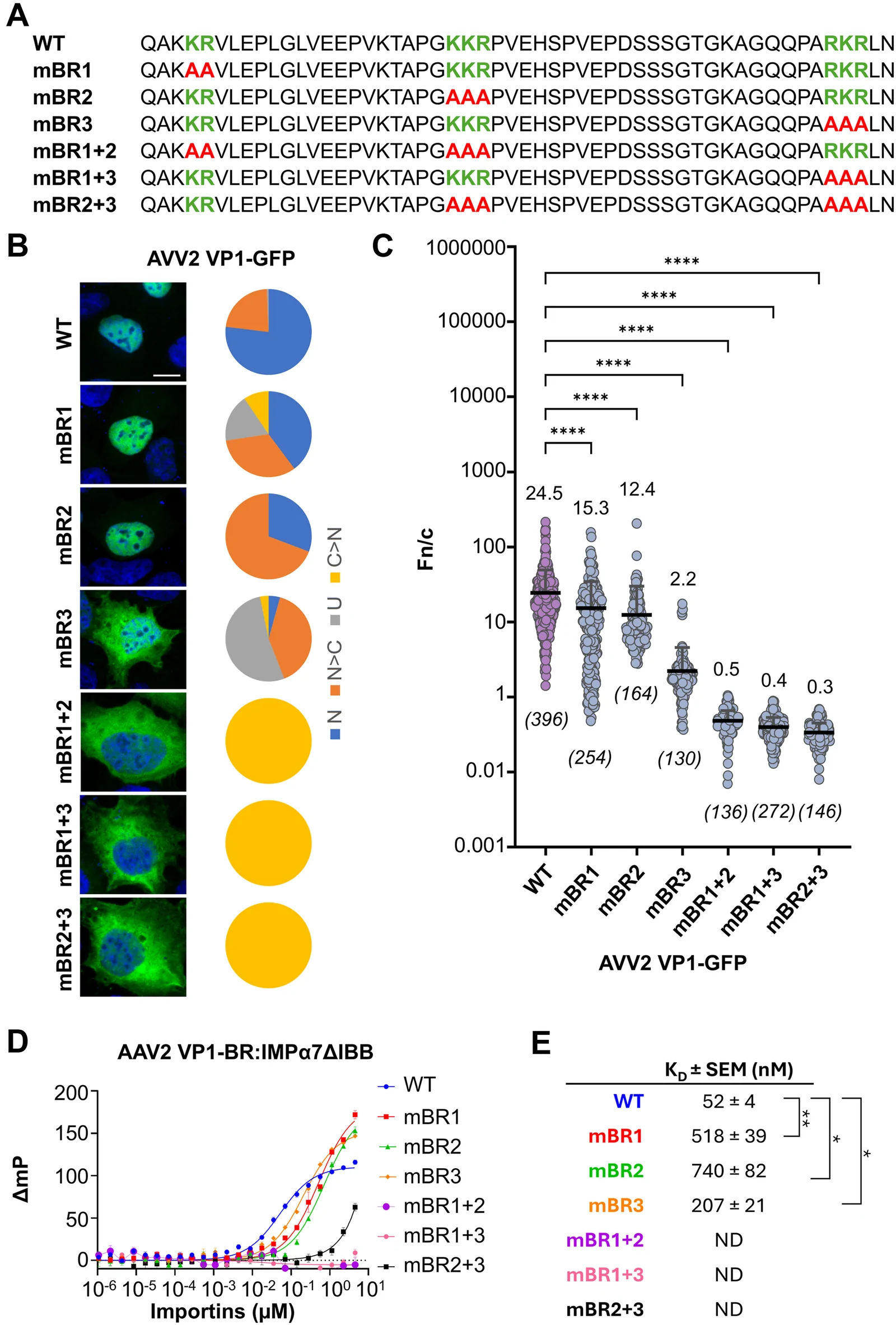
**Each BR within AAV2 VP1 N-terminus contributes to AAV2 VP1 nuclear targeting and interaction with IMPα.***A*, schematic overview of AAV2 VP1 BR1-3 substitution mutants. Unmodified BRs are shown in *green*, whereas basic residues substituted with alanine are shown in *red*. *B*, HEK293A cells were transfected with the indicated G FP-fusion constructs for 24 h, incubated with DRAQ5 to label nuclei, fixed and mounted for quantitative CLSM analysis. Representative merged images of cells expressing each construct are shown. Single-cell measurements were used to calculate the nuclear to cytoplasmic fluorescence ratio (Fn/c) Pie charts indicate the proportion of cells displaying the following phenotypes. N (Fn/c ≥ 5), N > C (2≤ Fn/c < 5), U (1< Fn/c < 2), C > N (Fn/c < 1). A total of n = 396 cells were analyzed for WT, n = 254 cells were analyzed for AAV2 VP1-mBR1, n = 164 cells were analyzed for AAV2 VP1-mBR2, n = 130 cells were analyzed for AAV2 VP1-mBR3, n = 136 cells were analyzed for AAV2 VP1-mBR1+2, n = 272 cells were analyzed for AAV2 VP1-mBR1+3, and n = 146 cells were analyzed for AAV2 VP1-mBR2+3. The scale bar represents = 10 μm , *C*, quantification of nuclear accumulation. *Each circle* represents an individual cell measurement, *black horizontal bars* indicate the mean, and *gray* vertical *bars* indicate standard deviation. Data are from three independent experiments. Statistical significance was assessed using Welch and Brown-Forsythe ANOVA tests (∗∗∗∗: *p* < 0.0001). *D*, FP analysis of binding between FITC labelled AAV2 VP1-BR substitution peptides and IMPα7. Data represent mean ± SEM from three independent experiments. *E*, summary of binding affinities derived from (*D*), showing mean K_D_ values ± SEM for each mutant. Statistical significance was assessed using Welch and Brown-Forsythe ANOVA tests (∗: *p* ≤ 0.05. ∗∗: *p* ≤ 0.01). ND: not determined. CLSM, confocal laser scanning microscopy; FITC, fluorescein isothiocyanate; FP, fluorescence polarization; IMPα, importin-α.

### Flexible switching of BR1 and BR2 at the IMPα minor binding site supports efficient AAV2 VP1 nuclear import

Our biochemical and cellular analysis indicate that the BR2 segment of AAV2 VP1 (residues ^140^PGKKRP^145^) contributes to nuclear import. This observation suggests that, despite not being resolved in the electron density of the AAV2 VP1-BR crystal structure, BR2 may stabilize the interaction either through direct binding to IMPα or by facilitating an optimal conformation of BR1 and BR3. To distinguish between these possibilities, we determined crystal structures of AAV2 VP1-BR peptides bearing alanine substitutions in either BR1 (mBR1: K123A/R124A) or BR3 (mBR3: R168A/K169A/R170A) in complex with mIMPα2. High resolution structures were obtained for both complexes (Fig. 5), consistent with their retained nuclear localization in cells (Fig. 4, *A*–*C*), and measurable binding to IMPα7 (Fig. 4, *D* and *E*).

**Figure 5:**
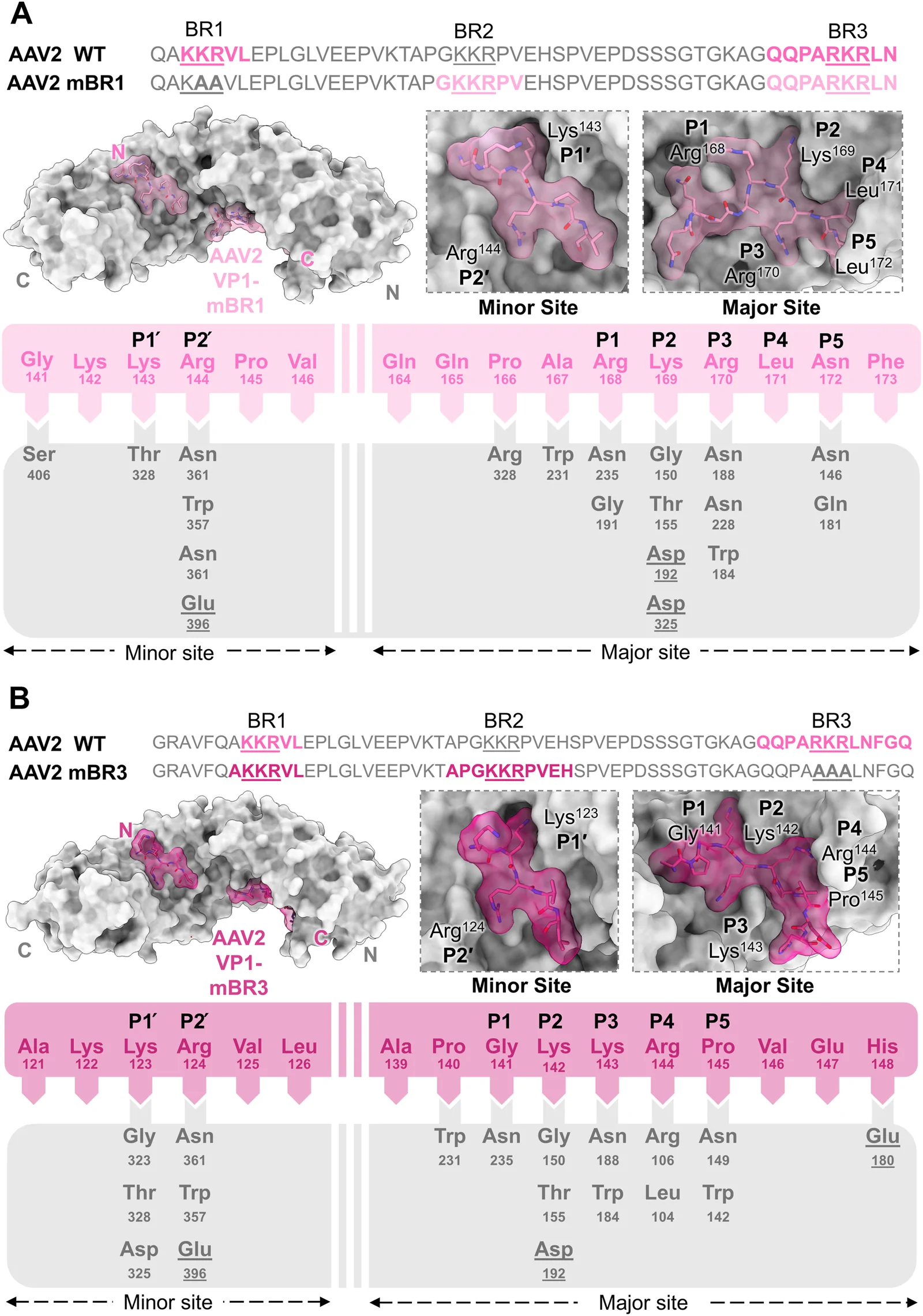
**BR2 of AAV2 VP1 can be accommodated at either the minor and major mIMPα2 binding site in a bipartite arrangement.***A*, sequence alignment of AAV2 VP1-BR and the mBR1 mutant, with residues resolved in the corresponding mIMPα2 complex highlighted in *pink*. Crystal structure of the AAV2 VP1-mBR1 (*pink sticks* and semi-transparent surface) bound to mIMPα2 (*gray surface*), resolved at 2.6 Å. Schematic representation of the interactions between AAV2 VP1-mBR1 and mIMPα2, including hydrogen bonds and salt bridges (*underlined*) as calculated using PDBsum. The structure has been deposited in the PDB under accession code 23QC. *B*, sequence alignment of AAV2 VP1-BR and the mBR3 mutant, with resolved residues in the mIMPα2 complex highlighted in *pink*. Crystal structure of the AAV2 VP1-mBR3 peptide (*pink sticks and semi*-*transparent surface*) bound to mIMPα2 (*gray surface*), resolved at 2.1 Å. Schematic representation of the interactions between AAV2 VP1-mBR3 and mIMPα2, including hydrogen bonds and salt bridges (*underlined*), as calculated using PDBsum. The structure has been deposited in the PDB under accession code 23QF. IMPα, importin-α; PDB, Protein Data Ban; PDBsum, Protein Data Bank sum.

In the AAV2 VP1-mBR1:mIMPα2 complex, BR3 occupies the major binding site, as observed for the WT peptide. Lys169 engages the P2 position, forming hydrogen bonds with Asp192, Thr155, and Gly150, and a salt bridge with Asp192. Notabley, BR2 is resolved in the minor binding site (Fig. 5*A*), where Lys143 forms hydrogen bonds with Val321, Gly323 and Thr328 (P1′ pocket), while Arg144 interacts with Trp357, Ser360 and Asn361, and forms a salt bridge with Glu396 (P2′ pocket). Conversely, in the AAV2 VP1-mBR3:mIMPα2 complex, BR2 occupies the major binding site, where Lys143 forms hydrogen bonds with Gly150, Thr155, and Asp192, and salt bridge interactions with Asp192 at the P2 position. In this configuration, the minor binding site is occupied by BR1, analogous to the WT structure (Fig. 5*B*). The electron density supporting the newly resolved BR2 regions in both mutant structures is shown in Fig. S6.

These findings demonstrate that distinct BR segments can engage alternative binding sites on IMPα, expanding the repertoire of transport competent VP1:IMPα complexes. To further evaluate this flexibility, we performed FoldX based binding free energy calculations using both experimental structures and derived models (Tables 1 and S5). Complexes containing BR1 or BR2 at the minor site and BR3 at the major site exhibited compared binding energies (−32.82 and −32.07 kj/mol, respectively), both more favorable than configurations with BR1 at the minor site and BR2 at the major site (−26,05 kj/mol). To systematically assess the compatibility of individual BR segments with each binding site, we performed computational peptide transposition and interaction energy analyses across available structural conformations. These analyses showed that both BR1 and BR2 can form favorable interactions at the minor binding site, whereas BR2 exhibits stronger compatibility with the major binding site (Tables 1 and S5–S9). Although BR3 can, in principle, form energetically favorable interactions at the minor site (Tables 1 and S10), its C-terminal position relative to BR1 and BR2 precludes its participation in a bipartite configuration at that site. In contrast, BR3 consistently forms highly stable interactions at the major binding site, with strongly favorable interaction energies ranging from −21.35 to −24.55 kcal/mol (Tables 1 and S11). Together, these results demonstrate that AAV2 VP1 can engage IMPα through multiple bipartite configurations, driven by the intrinsic flexibility of the BR region and the ability of BR2 to functionally substitute at both minor and major binding sites.

**Table 1:** Interaction energy calculations at the AAV2 VP1-BR1-3:mIMPα2 protein-protein interface derived from crystal structures and computational models

Interaction nteraction energy (kcal/mol)			
Crystal structures			
	Crystal 1: 23QB	Crystal 2: 23QC	Crystal 3: 23QF
	−36.84	−33.17	−26.05
Hybrid models of individual VP1 BRs			
BR1 minor site	−11.37^a^	−3.34^b^	−11.43^a^
BR1 major site	−0.99^b^	−1.27^b^	−10.03^b^
BR2 minor site	−2.89^b^	−11.3^a^	−9.71^b^
BR2 major site	−13.49^b^	−5.4^b^	−15.7^a^
BR3 minor site	−5.8^b^	−5.4^b^	−17.28^b^
BR3 major site	−21.35^a^	−23.19^a^	−24.55^b^

Interaction energies for indicated BR:mIMPα2 complexes are reported in kcal/mol.

a Values annotated with are derived from corresponding crystal structures.

b Values annotated with are obtained from hybrid models generated as described in Tables S6–S11.

## Discussion

The efficacy of AAVs as gene therapy vectors relies on their ability to deliver genetic material from the cytoplasm into the nucleus of host cells. Despite the central importance of this step, the molecular mechanisms underlying AAV nuclear entry, and in particular, the interactions between AAV capsids and host nuclear import machinery, remain incompletely understood. Previous studies have suggested a role of IMPβ1 in AAV nuclear import (31), however, the contribution of IMPα has remained largely unresolved in human AAV isolates, with the exception of our recent work demonstrating interactions between AAV6 and AAV11 VP1 and multiple IMPα paralogs (44, 45). Since its discovery in 1965 (1), AAV2 has become the most extensively studied serotype, both as a gene therapy vector and as a model for AAV biology. It is there notable that the precise mechanisms governing its nuclear entry have remained unclear. In this study, we provide a comprehensive functional, biochemical, and structural characterization of AAV2 VP1 interactions with IMPα isoforms. Using the competitive inhibitor Bimax2, which disrupts the classical IMPα/IMPβ1 nuclear import pathway (49), we demonstrate that nuclear import of full-length AAV2 VP1 is strongly dependent on IMPα (Fig. 2) These findings are consistent with our previous observations for AAV6 and AA11 (44, 45), supporting a conserved role for IMPα/β1 pathway in AAV nuclear trafficking.

Our finding that BR1-3 are sufficient to mediate interaction with multiple IMPα paralogs and to confer nuclear localization to heterologous proteins (Fig. 1) supports the hypothesis that VP1 is translocated into the nucleus *via* the classical nuclear import pathway. These results are consistent with previous studies implicating BRs within the N-terminal domain of AAV VP1 in nuclear import (30, 55). In the assembled capsid, where VP1, VP2, and VP3 are present in an approximate 1:1:10 ratio, the N-terminal domain of VP1 remains flexible and is largely internalized, becoming transiently exposed through capsid pores prior to nuclear import. Although these studies were performed using isolated VP1 BR peptides and recombinant VP1 proteins, there is currently no experimentally resolved structure of the flexible VP1/VP2 N-terminal region within an intact AAV capsid. Nevertheless, following endosomal trafficking this region becomes transiently externalized through the capsid pores, making the BR motifs accessible for interaction with host nuclear import receptors immediately prior to nuclear transport.

Building one these observations, our structural analyses indicates that AAV2 VP1 engages IMPα through a bipartite NLS arrangement in which different BR segments can occupy the canonical binding sites. The crystal structure of AAV2 VP1-BR:mIMPα2 complex revealed BR1 and BR3 positioned at the minor and major binding sites, respectively (Fig. 3). However, mutational analyses uncovered two unexpected features. First, substitution of basic residues within either BR1 or BR3, despite their involvement in key interactions with mIMPα2 structure, was insufficient to abolish IMPα binding (Fig. 4, *D* and *E*) or nuclear targeting (Fig. 4, *A*–*C*). This contrasts with the canonical model of bipartite NLSs, in which both basic clusters are typically required for functional binding and nuclear import (56). Secondly, mutation of BR2 significantly impaired both IMPα binding and nuclear localization (Fig. 4), despite BR2 not being resolved in the crystal structure (Fig. 3). Importantly, simultaneous mutation of any two BRs completely abrogated both IMPα binding and nuclear import (Fig. 4), indicating that AAV2 VP1 can utilize multiple combinations of BR segments to form a functional bipartite NLS. This model is strongly supported by structural data from mutant complexes (Fig. 5), which show that disruption of BR1 or BR3 allows BR2 to occupy the minor (Fig. 5*A*) or major (Fig. 5*B*) binding site, respectively. To our knowledge, this is the first demonstration that a viral bipartite NLS can maintain nuclear import rather than a single pair of basic motifs. Rather than functioning as a conventional bipartite NLS, AAV2 VP1 possesses a modular architecture in which alternative BR combinations can generate transport-competent IMPα complexes.

Together with *in silico* analyses, these findings demonstrate that BR1 and BR2 can functionally substitute for one another at the IMPα minor binding site (Tables 1 and S5–S11), highlighting the structural plasticity of the VP1:IMPα interface. Free energy calculations further support this model: when the major binding site is occupied by BR3, engagement of the minor site by either BR1 or BR2 yields comparable binding energies (−36.84 and −33.17 kJ/mol, respectively, see Table 1). This minimal energetic difference is consistent with the similar effects observed upon mutation of BR1 or BR2 on VP1 nuclear import (Fig. 4). These energetic analyses provide an explanation for why mutation of BR1 or BR2 individually has only modest functional consequences despite altering the structural arrangement of the IMPα interface.

Overall, our data support a model in which the VP1 N-terminal region possesses substantial conformational flexibility, allowing multiple transport competent bipartite NLS configurations to form. This interpretation is consistent with recent hydrogen deuterium exchange mass spectrometry studies demonstrating extensive flexibility within the VP1 N-terminus (57). Importantly, our findings also provide the first mechanistic explanation for how AAV2 VP2, which naturally lacks the BR1 containing N-terminal extension, can nevertheless undergo efficient nuclear import. The demonstration that the BR2-BR3 combination is sufficient to mediate productive IMPα recognition indicates that VP2 retains a fully functional bipartite NLS despite lacking BR1. Thus, the modular organization of BR1-3 is not simply a source of redundancy within VP1 but also provides a mechanistic framework explaining nuclear targeting of multiple AAV capsid proteins.

Comparison of AAV2 with previously characterized serotypes highlights both conservation and diversification of the AAV nuclear import mechanism. AAV2, AAV6 and AAV11 all utilize the classical IMPα/β1 pathway and employ VP1 BRs to engage IMPα paralogs, indicating that IMP-dependent nuclear transport is likely conserved across the genus. (44, 45). Structural comparison further reveals that AAV2 and AAV6 share a similar overall bipartite binding arrangement; with BR1 occupying the IMPα minor binding site and BR3 occupies the major binding site. Notably, the BR1 sequence and its interactions with the minor binding site are conserved between AAV2 and AAV6, whereas the BR3 sequence differs, with AAV2 containing a RKR motif and AAV6 contains a KKR motif within the major binding site (44). Thus, the conservation of the overall binding architecture despite variation in individual basic residues suggests that IMPα recognition can tolerate sequence variation while maintaining a functionally conserved bipartite arrangement. In contrast, AAV11 retains nuclear localization following disruption of BR1 and BR3 due to the presence of an additional BR between BR2 and BR3 (45). Together these observations suggest that AAV serotypes have evolved distinct but functionally convergent structural solutions to achieve efficient IMP recognition.

Collectively, these findings establish that AAV2 VP1 does not possess a single canonical bipartite NLS but instead utilizes a flexible, modular nuclear localization architecture capable of adopting multiple transport competent conformations. To our knowledge, this represents the first demonstration that alternative combinations of BRs can support efficient IMP mediated nuclear import within a viral bipartite NLS. More broadly, our work provides a structural framework for understanding nuclear trafficking of AAV capsid proteins and offers new opportunities to engineer capsid nuclear targeting by modulating individual BRs rather than introducing entirely new localization signals. Future studies should investigate how these alternative NLS arrangements influence nuclear entry of intact virions and whether similar modular architectures are conserved across additional AAV serotypes.

## Experimental procedures

### Expression plasmids

The amino acid sequence of AAV2 VP1 was obtained from the GenBank/Uniprot databases (accession number: P03135). A gene fragment corresponding to residues 115 to 175 of VP1, encompassing BRs BR1-3, (sequence: GRAVFQAKKRVLEPLGLVEEPVKTAPGKKRPVEHSPVEPDSSSGTGKAGQQPARKRLNFGQ), hereafter referred to as AAV2 VP1-BR, was codon optimized for expression in *E. coli.* The construct included an N-terminal glutathione S-transferase (GST) tag followed by a tobacco etch virus (TEV) cleavage site. The gene was cloned into the pGEX4T-1 vector (GenScript) at BamHI restriction sites. IMPα constructs used for crystallization, fluorescence polarization (FP), and EMSAs lacked the N-terminal IMPβ1 binding (IBB) domain (ΔIBB). The following constructs were used: human IMPα1(hIMPα1ΔIBB; His tag, TEV site), human IMPα3 (hIMPα3ΔIBB; His tag, TEV site), human IMPα5 (hIMPα5ΔIBB; His tag, TEV site), human IMPα7 (hIMPα7ΔIBB; His tag, TEV site), and mouse mIMPα2 (mIMPα2ΔIBB; His tag, no TEV site). All IMPα constructs were encoded in the pET-30a(+) vector and optimized for expression in *E. coli.* In addition, human IMPβ1 (hIMPβ1; HIS tag, TEV site) encoded in the pMCSG21 vector was used. These IMP constructs have been described previously (54, 58, 59).

Mammalian expression plasmids encoding either N-terminal GFP fusions of the AAV2 VP1 BR domain (AAV2 VP1-BR) or C-terminal GFP fusions of full length AAV2 VP1 protein (including substitution variants) were synthesized by BioFab Research. Control plasmids included pEPI-GFP-HCMV-UL44 and pEGFP-N1-H1E, express GFP fusion proteins that localize to the nucleus *via* IMPα/β1 dependent and independent pathways respectively (51, 60, 61). The plasmid mCherry-Bimax2, encoding a competitive inhibitor of the IMPα/β1 nuclear import pathway (62) was generously provided by Yoshihiro Yoneda and Masahiro Oka. Additional constructs included plasmid pcDNA3.1-NT-GFP-TOPO and pcDNA3.1-NT-GFP-TOPO-SV40-NLS, encoding a fusion between GFP cycle3, and the simian vacuolating virus 40 (SV40) LTA NLS (PKKKRKV-132) as described previously (63).

A complete list of plasmids used in this study is provided in Table S1.

### Fluorescein isothiocyanate labelled peptide design

Synthetic peptides corresponding to the AAV2 VP1-BR region were obtained from GenScript and labelled at the N-terminus with FITC *via* an aminohexanoic acid linker. Peptides were designed for the WT sequence and for variants in which basic residues within BR1, BR2, BR3 or combinations thereof (BR1+2, BR2+3, and BR1+3) were substituted with alanine. All peptides spanned residues 120 to 170 of AAV2 VP1. A completed list of FITC-labelled peptides used in this study is provided in Table S2.

### Recombinant bacterial protein expression and purification

Expression and co-purification of AAV2 VP1-BR with mIMPα2 were performed as described previously (46). AAV2 VP1-BR and mIMPα2 were individually overexpressed in *E. coli* pLysS cells using auto-induction method (64) at 23 °C, with shaking at 150 rpm for 30 to 38 h. Cells were harvested by centrifugation at 6000 rpm for 30 min at 16 °C and resuspended in the appropriate buffers; His buffer A for mIMPα2 (50 mM phosphate buffer, 300 mM NaCl, and 20 mM imidazole, pH 8.0), and GST buffer A for AAV2 VP1-BR (50 mM Tris and 125 mM NaCl). Cell suspensions were stored at −20 °C. Following two freeze-thaw cycles, cells were treated with lysozyme (50 mg/ml; #L6876, Sigma-Aldrich, USA) and DNAse (20 mg/ml; #DN25, Sigma-Aldrich) and incubated at room temperature for 1 h. Lysates were clarified by centrifugation 12,000 rpm for 45 min at 4 °C and filtered through a 0.45 μm low protein affinity filter. Soluble extracts of AAV2 VP1-BR with mIMPα2 were combined to allow complex formation and subsequently loaded onto a 5 ml HisTrap HP column (#17524801, Cytiva), pre-equilibrated with His buffer A, and bound proteins were eluted using a linear imidazole gradient (20–500 mM). Eluted fractions were pooled and incubated overnight at 4 °C with TEV protease to remove the GST tag from AAV2 VP1-BR. The sample was then subjected to size-exclusion chromatography using a HiLoad 26/600 Superdex 200 column (#28989336, Cytiva) equilibrated in GST buffer A. Fractions were collected and analyzed using SDS-PAGE (4–12% Bis-Tris Plus gel, #NW04120BOX, Thermo Fisher Scientific) at 165 V for 30 min. Protein samples were concentrated using a 10 kDa molecular weight cut-off centrifugal filter (#UFC9010, Merck Millipore).

Recombinant human proteins (IMPα1, mIMPα2, IMPα3, IMPα5, IMPα7, and IMPβ1) used for FP, and EMSAs were expressed and purified as described previously (43, 64). These proteins were produced using the same auto-induction conditions described above, however, purification was performed without prior complex formation, and His tags were not removed by TEV protease.

### Crystallization, data collection and structure determination of AAV2 VP1-BR:mIMPα2 complexes

All protein complexes were crystalized using the hanging drop vapor diffusion method (65), with protein and reservoir solutions mixed in a 1:1 ratio and incubated at 22 °C. Crystals were cryoprotected in reservoir solution supplemented with 20% (v/v) glycerol, prior to flash cooling in liquid nitrogen.

The AAV2 VP1-BR:mIMPα2 complex crystalized in a condition 0.7 M sodium citrate, 0.01 M DTT, 0.1 M Hepes (pH 6.5), yielding rod-shaped crystals after 2 days. For peptide bound complexes, AAV2 VP1-mBR1 and AAV2 VP1-mBR3 peptides were mixed with purified mIMPα2 at a 3:1 M ratio prior to crystallization. The AAV2 VP1-mBR1:mIMPα2 complex crystalized in 0.7 M sodium citrate, 0.01 M DTT, 0.1 M Hepes (pH 7.5), while the AAV2 VP1-mBR3:mIMPα2 complex crystalized under similar conditions at pH 7.0.

X-ray diffraction data were collected at the Australian Synchrotron on the MX2 beamline (66). Diffraction datasets were indexed and merged using DIALS (67), followed by scaling and merging with AIMLESS (68, 69). Structures were solved by molecular replacement in Phaser (70) using the Protein Data Bank (PDB) entry 4OIH (54) as the search model. Model building and refinement were performed iteratively using Coot (71, 72, 73) and Phenix (74). Final refinement statistics and stereochemical parameters are summarized in Table S3. Protein-peptide interactions, including hydrogen bonds and salt bridges, were analyzed using PDBsum database (75), with detailed interaction data provided in Table S4.

### EMSA

Synthetic FITC-labelled peptides (Table S2) were used at a final concentration of 10 μM and incubated with 20 μM of each IMP protein (IMPα1, mIMPα2, IMPα3, IMPα5, IMPα7 and IMPβ1), Samples were supplemented with 3 μl of 50% (v/v) glycerol. Samples were resolved on 1.5% agarose TB gel (45 mM Tris base, 45 mM Boric Acid, pH 8.5) and electrophoresed for 1.5 h at 70 V in TB buffer (45 mM Tris, 45 mM Boric Acid, pH 8.5). Gels were imaged using BioRad Gel Doc system with appropriate fluorescence filter. Following fluorescence imaging, gels were stained with with Coomassie Brilliant Blue R-250 staining solution (10% v/v ethanol, 10% v/v glacial acetic acid, 0.2% w/v Coomassie Brilliant Blue R-250) for 5 min, destained overnight in 10% (v/v) ethanol, 10% (v/v) glacial acetic acid, and imaged again using the BioRad Gel Doc imaging system.

### FP assay

FITC labelled peptides (Table S2) were incubated at a final concentration of 2 nM with two-fold serially dilutions of IMP proteins (IMPα1, mIMPα2, IMPα3, IMPα5, IMPα7 and IMPβ1), starting from 4.5 μM across 23 wells to a final volume of 200 μl per well in GST buffer A (50 mM Tris, 125 mM NaCl). The 24th well contained peptide alone and was used to set the instrument gain. FP measurements were recorded and analyzed as described previously (43). All assays were performed in three independent experiments, each including an appropriate negative control (no IMP). Data were analyzed using GraphPad Prism (version 9.3.1; https://www.graphpad.com/features), and binding curves were fitted to determine dissociation constant (K_D_).

### Molecular modelling

Molecular modelling and interaction energy calculations of AAV2 VP1-BR:mIMPα2 peptide complexes were performed using the FoldX 5.1 suite accessed *via* the YASARA graphical interface (76, 77). The structural stability and interaction energies of mIMPα2ΔIBB in complex with synthetic AAV2 VP1-BR peptides were analyzed. Prior to energy calculations, all crystal structures were energy minimized using the YASARA Minimization server and optimized with the RepairPDB function to correct local steric clashes and structural imperfections. Binding energies of different BR combinations (BR1+2, BR1+3 and BR2+3) as observed in the crystal structures occupying the minor and major binding sites of mIMPα2, were then calculated using the FoldX AnalyseComplex command with default parameters. To further quantify the contribution of individual BRs, residues corresponding to the non-target BR in each AAV2 VP1-BR:mIMPα2 crystal structure were removed using PyMOL (77). In addition, hybrid models were generated to evaluate binding of each BR at either the major or minor binding site. These models were constructed by superimposing the relevant BR peptide into the corresponding binding site using the crystallographic complex as a reference, followed by removal of non-relevant BR regions. All generated all-atom models were subsequently energy-minimized using the YASARA Minimization server and re-optimized with RepairPDB prior to analysis (76, 77, 78).

### Cell culture and transfections

HEK293A (#R70507; Thermo Fisher Scientific) cells were maintained in Dulbecco's modified Eagle's medium (DMEM) supplemented with 10% (v/v) foetal bovine serum, 50 U/ml penicillin, 50 U/ml streptomycin, and 2 mM L-glutamine. Cells were culture in a humidified incubator at 37 °C with 5% CO_2_ and passaged upon reaching confluency. For transfection, HEK293A cells were seeded onto glass coverslips in 24-well plates at a density of 5 × 10^4^ cells/well. The following day, cells were transfected with the indicated expression constructs (range 5–250 ng per well), using Lipofectamine 2000 (Thermo Fisher Scientific) according to the manufacturer’s instructions (79). Cells were then incubated under standard culture conditions until analysis by CLSM.

### CLSM and image analysis

Twenty four h post-transfection, cells were incubated with DRAQ5 (#62251, Thermo Fisher Scientific; 1:5000 in DMEM no phenol red) for 30 min. Cells were then washed twice with PHEM 1x (60 mM PIPES, 25 mM Hepes, 10 mM EGTA, and 4 mM MgSO_4_) and fixed with 4% (v/v) paraformaldehyde in PHEM buffer for 10 min at room temperature. Following fixation, cells were washed three times 1x PBS and mounted on glass slides using Fluoromount G (Southern Biotech). Subcellular localization of fluorescent fusion proteins was analyzed using a Nikon A1 confocal laser scanning microscope (Nikon) equipped with a 60x oil immersion objective, as described previously (80). Nuclear accumulation was quantified using Fiji software (81) by measuring single-cell nuclear (Fn), and cytoplasmic (Fc) fluorescence intensities following subtraction of background/autofluorescence, as described previously (82). Individual cells were analyzed across at least three independent experiments, with the number of cells analyzed for each experimental condition reported in the corresponding figure panels. Cells with oversaturated signals were excluded from the analysis; Fn/c ratios exceeding 255 were considered outliers and excluded from the analysis. Statistical analysis was performed using Welch’s and Brown-Forsythe ANOVA tests in GraphPad Prism (version 9.3.1, GraphPad).

## Data availability

Maps and model for AAV2 VP1-BRwt in complex with mIMPα2 have been deposited to the Protein Data Bank, under accession code 23QB. Maps and model for AAV2 VP1-mBR1 in complex with mIMPα2 have been deposited to the Protein Data Bank, under accession code 23QC. AAV2 VP1-mBR3 in complex with mIMPα2 has been deposited to the Protein Data Bank, under accession code 23QF.

## Supporting information

This article contains supporting information (51, 62, 63).

## Conflict of interest

The authors declare that they have no conflicts of interest with the contents of this article.

## References

[1] Atchison R.W., Casto B.C., Hammon W.M. (1965). Adenovirus-associated defective virus particles. Science.

[2] Chowdhury E.A., Meno-Tetang G., Chang H.Y., Wu S., Huang H.W., Jamier T. (2021). Current progress and limitations of AAV mediated delivery of protein therapeutic genes and the importance of developing quantitative pharmacokinetic/pharmacodynamic (PK/PD) models. Adv. Drug Deliv. Rev..

[3] Cotmore S.F., Agbandje-McKenna M., Canuti M., Chiorini J.A., Eis-Hubinger A.M., Hughes J. (2019). ICTV virus taxonomy profile: parvoviridae. J. Gen. Virol..

[4] Cotmore S.F., Agbandje-McKenna M., Chiorini J.A., Mukha D.V., Pintel D.J., Qiu J. (2014). The family parvoviridae. Arch. Virol..

[5] Wang D., Tai P.W.L., Gao G. (2019). Adeno-associated virus vector as a platform for gene therapy delivery. Nat. Rev. Drug Discov..

[6] Kuz C.A., McFarlin S., Qiu J. (2024). The expression and function of the small nonstructural proteins of adeno-associated viruses (AAVs). Viruses.

[7] Li C., Samulski R.J. (2020). Engineering adeno-associated virus vectors for gene therapy. Nat. Rev. Genet..

[8] Wu Z., Asokan A., Samulski R.J. (2006). Adeno-associated virus serotypes: vector toolkit for human gene therapy. Mol. Ther..

[9] Schmidt M., Voutetakis A., Afione S., Zheng C., Mandikian D., Chiorini J.A. (2008). Adeno-associated virus type 12 (AAV12): a novel AAV serotype with sialic acid- and heparan sulfate proteoglycan-independent transduction activity. J. Virol..

[10] Lin D.H., Hoelz A. (2019). The structure of the nuclear pore complex (an update). Annu. Rev. Biochem..

[11] Liu Q., Huang W., Zhang H., Wang Y., Zhao J., Song A. (2014). Neutralizing antibodies against AAV2, AAV5 and AAV8 in healthy and HIV-1-infected subjects in China: implications for gene therapy using AAV vectors. Gene Ther..

[12] Louis Jeune V., Joergensen J.A., Hajjar R.J., Weber T., Liu Q., Huang W. (2013). Pre-existing anti-adeno-associated virus antibodies as a challenge in AAV gene therapy. Hum. Gene Ther. Methods.

[13] Berns K.I., Muzyczka N. (2017). AAV: an overview of unanswered questions. Hum. Gene Ther..

[14] Smith R.H. (2008). Adeno-associated virus integration: virus versus vector. Gene Ther..

[15] Atchison R.W. (1970). The role of herpesviruses in adenovirus-associated virus replication in vitro. Virology.

[16] Berns K., P.C R., Knipe D.M., Howley P.M. (2013). Fields Virology.

[17] Ogston P., Raj K., Beard P. (2000). Productive replication of adeno-associated virus can occur in human papillomavirus type 16 (HPV-16) episome-containing keratinocytes and is augmented by the HPV-16 E2 protein. J. Virol..

[18] Asokan A., Conway J.C., Phillips J.L., Li C., Hegge J., Sinnott R. (2010). Reengineering a receptor footprint of adeno-associated virus enables selective and systemic gene transfer to muscle. Nat. Biotechnol..

[19] Flotte T.R., Afione S.A., Zeitlin P.L. (1994). Adeno-associated virus vector gene expression occurs in nondividing cells in the absence of vector DNA integration. Am. J. Respir. Cell Mol. Biol..

[20] Grimm D., Pandey K., Nakai H., Storm T.A., Kay M.A. (2006). Liver transduction with recombinant adeno-associated virus is primarily restricted by capsid serotype not vector genotype. J. Virol..

[21] Wu P., Phillips M.I., Bui J., Terwilliger E.F. (1998). Adeno-associated virus vector-mediated transgene integration into neurons and other nondividing cell targets. J. Virol..

[22] Zincarelli C., Soltys S., Rengo G., Rabinowitz J.E. (2008). Analysis of AAV serotypes 1-9 mediated gene expression and tropism in mice after systemic injection. Mol. Ther..

[23] Hirsch M.L., Wolf S.J., Samulski R.J. (2016). Delivering transgenic DNA exceeding the carrying capacity of AAV vectors. Methods Mol. Biol..

[24] Nieuwenhuis B., Laperrousaz E., Tribble J.R., Verhaagen J., Fawcett J.W., Martin K.R. (2023). Improving adeno-associated viral (AAV) vector-mediated transgene expression in retinal ganglion cells: comparison of five promoters. Gene Ther..

[25] Dhungel B.P., Bailey C.G., Rasko J.E.J. (2021). Journey to the center of the cell: tracing the path of AAV transduction. Trends Mol. Med..

[26] Hansen J., Qing K., Srivastava A. (2001). Infection of purified nuclei by adeno-associated virus 2. Mol. Ther..

[27] Kelich J.M., Ma J., Dong B., Wang Q., Chin M., Magura C.M. (2015). Super-resolution imaging of nuclear import of adeno-associated virus in live cells. Mol. Ther. Methods Clin. Dev..

[28] Milles S., Mercadante D., Aramburu I.V., Jensen M.R., Banterle N., Koehler C. (2015). Plasticity of an ultrafast interaction between nucleoporins and nuclear transport receptors. Cell.

[29] Xiao P.J., Samulski R.J. (2012). Cytoplasmic trafficking, endosomal escape, and perinuclear accumulation of adeno-associated virus type 2 particles are facilitated by microtubule network. J. Virol..

[30] Grieger J.C., Snowdy S., Samulski R.J. (2006). Separate basic region motifs within the adeno-associated virus capsid proteins are essential for infectivity and assembly. J. Virol..

[31] Nicolson S.C., Samulski R.J. (2014). Recombinant adeno-associated virus utilizes host cell nuclear import machinery to enter the nucleus. J. Virol..

[32] McPherson A.J., Lange A., Doetsch P.W., Corbett A.H. (2015). Encyclopedia of life sciences.

[33] Lange A., Mills R.E., Lange C.J., Stewart M., Devine S.E., Corbett A.H. (2007). Classical nuclear localization signals: definition, function, and interaction with importin alpha. J. Biol. Chem..

[34] Lu J., Wu T., Zhang B., Liu S., Song W., Qiao J. (2021). Types of nuclear localization signals and mechanisms of protein import into the nucleus. Cell Commun. Signal..

[35] Lange A., McLane L.M., Mills R.E., Devine S.E., Corbett A.H. (2010). Expanding the definition of the classical bipartite nuclear localization signal. Traffic.

[36] Becerra S.P., Koczot F., Fabisch P., Rose J.A. (1988). Synthesis of adeno-associated virus structural proteins requires both alternative mRNA splicing and alternative initiations from a single transcript. J. Virol..

[37] Becerra S.P., Rose J.A., Hardy M., Baroudy B.M., Anderson C.W. (1985). Direct mapping of adeno-associated virus capsid proteins B and C: a possible ACG initiation codon. Proc. Natl. Acad. Sci. U. S. A..

[38] Srivastava A., Lusby E.W., Berns K.I. (1983). Nucleotide sequence and organization of the adeno-associated virus 2 genome. J. Virol..

[39] Kronenberg S., Kleinschmidt J.A., Böttcher B. (2001). Electron cryo-microscopy and image reconstruction of adeno-associated virus type 2 empty capsids. EMBO Rep..

[40] Mietzsch M., Li Y., Kurian J., Smith J.K., Chipman P., McKenna R. (2020). Structural characterization of a bat Adeno-associated virus capsid. J. Struct. Biol..

[41] Kronenberg S., Böttcher B., von der Lieth C.W., Bleker S., Kleinschmidt J.A. (2005). A conformational change in the adeno-associated virus type 2 capsid leads to the exposure of hidden VP1 N termini. J. Virol..

[42] Stahnke S., Lux K., Uhrig S., Kreppel F., Hösel M., Coutelle O. (2011). Intrinsic phospholipase A2 activity of adeno-associated virus is involved in endosomal escape of incoming particles. Virology.

[43] Hoad M., Cross E.M., Donnelly C.M., Sarker S., Roby J.A., Forwood J.K. (2023). Structural characterization of porcine adeno-associated virus capsid protein with nuclear trafficking protein importin alpha reveals a bipartite nuclear localization signal. Viruses.

[44] Hoad M., Nematollahzadeh S., Petersen G.F., Roby J.A., Alvisi G., Forwood J.K. (2024). Structural basis for nuclear import of adeno-associated virus serotype 6 capsid protein. J. Virol..

[45] Hoad M., Nematollahzadeh S., Roby J.A., Alvisi G., Forwood J.K. (2025). Elucidation of the binding interaction interface between AAV serotype 11 capsid protein and host nuclear import proteins. Mol. Ther. Methods Clin. Dev..

[46] Hoad M., Roby J.A., Forwood J.K. (2021). Structural characterization of the porcine adeno-associated virus Po1 capsid protein binding to the nuclear trafficking protein importin alpha. FEBS Lett..

[47] Hoad M., Roby J.A., Forwood J.K. (2024). Structural basis for nuclear import of bat adeno-associated virus capsid protein. J. Gen. Virol..

[48] Au H.K.E., Isalan M., Mielcarek M. (2021). Gene therapy advances: a meta-analysis of AAV usage in clinical settings. Front. Med. (Lausanne).

[49] Kosugi S., Hasebe M., Entani T., Takayama S., Tomita M., Yanagawa H. (2008). Design of peptide inhibitors for the importin α/β nuclear import pathway by activity-based profiling. Chem. Biol..

[50] Jäkel S., Albig W., Kutay U., Bischoff F.R., Schwamborn K., Doenecke D. (1999). The importin beta/importin 7 heterodimer is a functional nuclear import receptor for histone H1. EMBO J..

[51] Alvisi G., Jans D.A., Guo J., Pinna A., Ripalti A. (2005). A protein kinase CK2 site flanking the nuclear targeting signal enhances nuclear transport of human cytomegalovirus ppUL44. Traffic.

[52] Kobe B. (1999). Autoinhibition by an internal nuclear localization signal revealed by the crystal structure of mammalian importin alpha. Nat. Struct. Mol. Biol..

[53] Pang X., Zhou H.X. (2014). Design rules for selective binding of nuclear localization signals to minor site of importin α. PLoS One.

[54] Roman N., Christie M., Swarbrick C.M.D., Kobe B., Forwood J.K. (2013). Structural characterisation of the nuclear import receptor importin alpha in complex with the bipartite NLS of Prp20. PLoS One.

[55] Popa-Wagner R., Sonntag F., Schmidt K., King J., Kleinschmidt J.A. (2012). Nuclear translocation of adeno-associated virus type 2 capsid proteins for virion assembly. J. Gen. Virol..

[56] Navarro M.V., Vierira D.F., Nagem R.A., de Araújo A.P., Oliva M.L., Garratt R.C. (2005). Crystallization and preliminary X-ray analysis of a novel Kunitz-type kallikrein inhibitor from Bauhinia bauhinioides. Acta Crystallogr. Sect. F Struct. Biol. Cryst. Commun..

[57] Ye X., Hu Y., Qiu H., Li N. (2024). Probe capsid structure stability and dynamics of adeno-associated virus as an important viral vector for gene therapy by hydrogen-deuterium exchange-mass spectrometry. Protein Sci..

[58] Munasinghe T.S., Edwards M.R., Tsimbalyuk S., Vogel O.A., Smith K.M., Stewart M. (2022). MERS-CoV ORF4b employs an unusual binding mechanism to target IMPα and block innate immunity. Nat. Commun..

[59] Teh T., Tiganis T., Kobe B. (1999). Crystallization of importin alpha, the nuclear-import receptor. Acta Crystallogr. D Biol. Crystallogr..

[60] Alvisi G., Avanzi S., Musiani D., Camozzi D., Leoni V., Ly-Huynh J.D. (2008). Nuclear import of HSV-1 DNA polymerase processivity factor UL42 is mediated by a C-terminally located bipartite nuclear localization signal. Biochemistry.

[61] Alvisi G., Ripalti A., Ngankeu A., Giannandrea M., Caraffi S.G., Dias M.M. (2006). Human cytomegalovirus DNA polymerase catalytic subunit pUL54 possesses independently acting nuclear localization and ppUL44 binding motifs. Traffic.

[62] Tsujii A., Miyamoto Y., Moriyama T., Tsuchiya Y., Obuse C., Mizuguchi K. (2015). Retinoblastoma-binding protein 4-regulated classical nuclear transport is involved in cellular senescence. J. Biol. Chem..

[63] Cross E.M., Akbari N., Ghassabian H., Hoad M., Pavan S., Ariawan D. (2023). A functional and structural comparative analysis of large tumor antigens reveals evolution of different importin α-dependent nuclear localization signals. Protein Sci..

[64] Studier F.W. (2005). Protein production by auto-induction in high-density shaking cultures. Protein Expr. Purif..

[65] Dessau M.A., Modis Y. (2011). Protein crystallization for X-ray crystallography. J. Vis. Exp..

[66] McPhillips T.M., McPhillips S.E., Chiu H.J., Cohen A.E., Deacon A.M., Ellis P.J. (2002). Blu-ice and the distributed control system: software for data acquisition and instrument control at macromolecular crystallography beamlines. J. Synchrotron Radiat..

[67] Winter G., Beilsten-Edmands J., Devenish N., Gerstel M., Gildea R.J., McDonagh D. (2022). DIALS as a toolkit. Protein Sci..

[68] Evans P. (2006). Scaling and assessment of data quality. Acta Crystallogr. D Biol. Crystallogr..

[69] Evans P.R. (2011). An introduction to data reduction: space-group determination, scaling and intensity statistics. Acta Crystallogr. D Biol. Crystallogr..

[70] McCoy A.J., Grosse-Kunstleve R.W., Adams P.D., Winn M.D., Storoni L.C., Read R.J. (2007). Phaser crystallographic software. J. Appl. Crystallogr..

[71] Emsley P., Cowtan K. (2004). Coot: model-building tools for molecular graphics. Acta Crystallogr. D Biol. Crystallogr..

[72] Emsley P., Lohkamp B., Scott W.G., Cowtan K. (2010). Features and development of coot. Acta Crystallogr. D Biol. Crystallogr..

[73] Winn M.D., Ballard C.C., Cowtan K.D., Dodson E.J., Emsley P., Evans P.R. (2011). Overview of the CCP4 suite and current developments. Acta Crystallogr. D Biol. Crystallogr..

[74] Adams P.D., Afonine P.V., Bunkoczi G., Chen V.B., Davis I.W., Echols N. (2010). PHENIX: a comprehensive Python-based system for macromolecular structure solution. Acta Crystallogr. D Biol. Crystallogr..

[75] Laskowski R.A., Thornton J.M. (2022). PDBsum extras: SARS-CoV-2 and AlphaFold models. Protein Sci..

[76] Buß O., Rudat J., Ochsenreither K. (2018). FoldX as protein engineering tool: better than random based approaches?. Comput. Struct. Biotechnol. J..

[77] Tragni V., Mercurio I., Paoletti D.P., Onofrio A., Laera L., Cafferati Beltrame L. (2023). Deconstructing SARS-CoV-2 neutralization: a modular molecular framework for computational design and comparison of antibodies and nanobodies targeting the spike RBD. J. Med. Virol..

[78] Krieger E., Joo K., Lee J., Lee J., Raman S., Thompson J. (2009). Improving physical realism, stereochemistry, and side-chain accuracy in homology modeling: four approaches that performed well in CASP8. Proteins.

[79] Di Antonio V., Palù G., Alvisi G. (2021). Live-cell analysis of human cytomegalovirus DNA polymerase holoenzyme assembly by resonance energy transfer methods. Microorganisms.

[80] Smith K.M., Di Antonio V., Bellucci L., Thomas D.R., Caporuscio F., Ciccarese F. (2018). Contribution of the residue at position 4 within classical nuclear localization signals to modulating interaction with importins and nuclear targeting. Biochim. Biophys. Acta Mol. Cell Res..

[81] Schindelin J., Arganda-Carreras I., Frise E., Kaynig V., Longair M., Pietzsch T. (2012). Fiji: an open-source platform for biological-image analysis. Nat. Methods.

[82] Alvisi G., Paolini L., Contarini A., Zambarda C., Di Antonio V., Colosini A. (2018). Intersectin goes nuclear: secret life of an endocytic protein. Biochem. J..

